# Impact of a Bacterial Volatile 2,3-Butanediol on *Bacillus subtilis* Rhizosphere Robustness

**DOI:** 10.3389/fmicb.2016.00993

**Published:** 2016-06-28

**Authors:** Hwe-Su Yi, Yeo-Rim Ahn, Geun C. Song, Sa-Youl Ghim, Soohyun Lee, Gahyung Lee, Choong-Min Ryu

**Affiliations:** ^1^Molecular Phytobacteriology Laboratory, Korea Research Institute of Bioscience and Biotechnology, DaejeonSouth Korea; ^2^School of Life Science, Kyungpook National University, DaeguSouth Korea; ^3^Department of Biological Science, Korea Advanced Institute of Science and Technology, DaejeonSouth Korea; ^4^Biosystems and Bioengineering Program, School of Science, University of Science and Technology, DaejeonSouth Korea

**Keywords:** PGPR, ISR, volatile, 2, 3-butanediol, bacteria robustness

## Abstract

Volatile compounds, such as short chain alcohols, acetoin, and 2,3-butanediol, produced by certain strains of root-associated bacteria (rhizobacteria) elicit induced systemic resistance in plants. The effects of bacterial volatile compounds (BVCs) on plant and fungal growth have been extensively studied; however, the impact of bacterial BVCs on bacterial growth remains poorly understood. In this study the effects of a well-characterized bacterial volatile, 2,3-butanediol, produced by the rhizobacterium *Bacillus subtilis*, were examined in the rhizosphere. The nature of 2,3-butanediol on bacterial cells was assessed, and the effect of the molecule on root colonization was also determined. Pepper roots were inoculated with three *B. subtilis* strains: the wild type, a 2,3-butanediol overexpressor, and a 2,3-butanediol null mutant. The *B. subtilis* null strain was the first to be eliminated in the rhizosphere, followed by the wild-type strain. The overexpressor mutant was maintained at roots for the duration of the experiment. Rhizosphere colonization by a saprophytic fungus declined from 14 days post-inoculation in roots treated with the *B. subtilis* overexpressor strain. Next, exudates from roots exposed to 2,3-butanediol were assessed for their impact on fungal and bacterial growth *in vitro*. Exudates from plant roots pre-treated with the 2,3-butanediol overexpressor were used to challenge various microorganisms. Growth was inhibited in a saprophytic fungus (*Trichoderma* sp.), the 2,3-butanediol null *B. subtilis* strain, and a soil-borne pathogen, *Ralstonia solanacearum*. Direct application of 2,3-butanediol to pepper roots, followed by exposure to *R. solanacearum*, induced expression of *Pathogenesis-Related* (*PR*) genes such as *CaPR2*, *CaSAR8.2*, and *CaPAL*. These results indicate that 2,3-butanediol triggers the secretion of root exudates that modulate soil fungi and rhizosphere bacteria. These data broaden our knowledge regarding bacterial volatiles in the rhizosphere and their roles in bacterial fitness and as important inducers of plant defenses.

## Introduction

The rhizosphere is defined as the narrow area surrounding the plant root system. Root exudates within the rhizosphere act as a food source for other organisms (Bowen and Rovira, 1999). As a result, the rhizosphere is an important habitat for many different microbes, and acts as a competitive arena for roots and soil-borne-pathogenic and rhizosphere bacteria (rhizobacteria; Vespermann et al., 2007). Among the rhizobacteria, plant growth-promoting rhizobacteria (PGPR) have been the subject of much research in recent decades. PGPR colonization of roots promotes plant growth and enhances crop yields (biostimulants), and can help protect against plant pathogens (bioprotectants; Kloepper and Metting, 1992; Kloepper et al., 2004; Ryu et al., 2004; Calvo et al., 2014; Chung et al., 2016). PGPRs act as biostimulants and bioprotectants by (1) acting antagonistically to target pathogens, (2) producing plant hormone mimics, and (3) inducing systemic resistance (Kloepper and Ryu, 2006).

Volatile compounds such as isoprene, terpenes, alkanes, alkenes, alcohols, esters, carbonyls, and acids can influence communication between organisms, including between bacteria and plants (Kesselmeier and Staudt, 1999; Ryu et al., 2005a; Kai et al., 2007). Previous research reported that PGPR bacilli emitted volatiles that triggered plant growth promotion and induced systemic resistance (ISR; Ryu et al., 2003, 2004; Chung et al., 2016). After this discovery, numerous studies identified further bacterial volatiles and determined their effects on plant responses. Some volatiles are now available for field applications (Cortes-Barco et al., 2010a,b; Farag et al., 2013; Chung et al., 2016). One well-characterized volatile is 2,3-butanediol, which was examined in multiple Gram-negative and Gram-positive bacterial species such as *Bacillus* spp., *Aerobacter* spp., *Serratia* spp., *Enterobacter* spp., and *Klebsiella* spp. (Barrett et al., 1983; Voloch et al., 1985; Ryu et al., 2004; Han et al., 2006). Acetoin is the last synthesis intermediate for 2,3-butanediol, and metabolic conversion of acetoin to 2,3-butanediol is reversible in most bacteria but irreversible in fungi such as yeast (Syu, 2001). Acetoin and 2,3-butanediol also mediate plant-beneficial effects such as growth promotion and ISR in model plants and crops under *in vitro* and *in situ* conditions (Ryu et al., 2003, 2004; Han et al., 2006; Hahm et al., 2012). Recent metabolic engineering approaches facilitated increased production of 2,3-butanediol in non-producer or low-producer bacterial species such as *Klebsiella oxytoca*, *Escherichia coli*, and *Paenibacillus polymyxa* by introduction of new genes and modification of biosynthetic pathways (Ji et al., 2011, 2014; Yang et al., 2013; Bai et al., 2015). However, the benefit to bacteria of producing 2,3-butanediol in the anaerobic conditions of the rhizosphere remains unknown.

The role of 2,3-butanediol in bacterial fitness has not been intensively studied. Early research in a mouse model revealed that 2,3-butanediol synthesis in *Vibrio cholerae* conferred a survival advantage *in vivo* during infection of intestines (Yoon and Mekalanos, 2006; Xiao and Xu, 2007). It is thought that 2,3-butanediol acts as a neutralizer in the acidic conditions of the intestinal cells. A null mutant that was unable to produce 2,3-butanediol was unable to colonize or maintain the bacterial populations during infection (Xiao and Xu, 2007; Pradhan et al., 2010; Bari et al., 2011). We hypothesized that 2,3-butanediol might play a similar bacterial fitness role in the rhizosphere. In this study, the effects of 2,3-butanediol on rhizosphere colonization were examined using three strains: *Bacillus subtilis* 168, BSIP1174 [a 2,3-butanediol null mutant referred to as “2,3-B(-)”], and BSIP1171 [an overexpression strain referred to as “2,3-B(+++)”]. In addition, the indirect effects of 2,3-butanediol on secretion of root exudates were examined in pepper roots. Finally, the antimicrobial capacity of root exudates elicited by 2,3-butanediol treatment was assessed. Exudates exhibited selective antagonism against pathogenic bacteria such as *Ralstonia solanacearum.* To our knowledge, this is the first report to characterize a bacterial volatile under *in situ* conditions in plants and to validate *in vitro*.

## Materials and Methods

### Plant Materials and Bacterial Preparation

Plants were grown were carried out as previously described (Kang et al., 2007). Briefly, seeds of *Capsicum annuum* were surface-sterilized with 6% sodium hypochlorite, washed four times with sterile distilled water (SDW), and then maintained at 25°C for 3 days until germination on Murashige and Skoog medium (Duchefa, Haarlem, the Netherlands). Germinated seeds were then transplanted to soilless media (Punong Horticulture Nursery Media LOW, Punong, Co. Ltd., Gyeongju, South Korea). Plants were grown at 25 ± 2°C under fluorescent light (12 h/12 h day/night cycle, 7000 lx light intensity) in a controlled-environment growth room. After establishment of seedlings, plants were transferred to the KRIBB greenhouse facility in Daejeon, South Korea.

Three *B. subtilis* strains were used to assess the role of 2,3-butanediol on bacterial rhizosphere competence: 168, BSIP1174 [2,3-butanediol null mutant referred to as 2,3-B(-)], and BSIP1171 [2,3-butanediol overexpression mutant referred to as 2,3-B(++); Cruz et al., 2000]. Bacterial suspension (5 ml at 10^8^ colony forming units/ml) was used to inoculate pepper roots, as described previously (Lee et al., 2012, 2013). A spontaneous rifampicin resistance mutant of wild-type *B. subtilis* 168 was isolated previously (Ryu et al., 2005b). Bacterial strains were isolated from plant roots using specific antibiotics in the tryptic soy broth agar growth medium (TSA, BactoTM, BD, Sparks, MD, USA): 50 μg/ml rifampicin for strain 168, 10 μg/ml spectinomycin for 2,3-B(-), and 10 μg/ml spectinomycin plus 5 μg/ml chloramphenicol for 2,3-B(++). The experiment was repeated three times with five replications (one plant per replication).

The naturally occurring soil fungus was isolated from dilution plating method of pepper root system when we attempted to assess *B. subtilis* population described above.

### Disease Assay of *Ralstonia solanacearum*

Spontaneous rifampicin resistant *R. solanacearum*, was grown on solid Casamino acid-Peptone-Glucose [CPG, 1 g casamino acid (casein hydrolysate), 10 g peptone, 5 g glucose, and 18 g agar per 1 L water] medium containing 100 μg/ml rifampicin at 30°C for 2 days, scraped off the plates, re-suspended in sterilized distilled water and adjusted to 10^8^ cfu/ml concentration for further experiments (Lee et al., 2012). The plants pretreated with 1 mM BTH was used a positive control. The 10 ml suspension of *R. solanacearum* was drenched on 3 weeks-old pepper seedlings at 1 week after 1 mM and 10 μM 2,3-butanediol and BTH drench-application as describe previously (Lee et al., 2012). To assess pathogen multiplication, the root sample at 0 and 3 days after pathogen challenge collected, macerated with sterile mortar and pestle, and plating on CPG agar medium containing 100 μg/ml rifampicin. The number of CFU was counted at 2–3 days after incaution of the plates.

### Assessment of Bacterial Populations Isolated from Pepper Roots

Bacterial colonization on roots was determined at 0, 7, 14, 21, and 28 days after treatment, as described previously (Ryu et al., 2005b). Briefly, root samples were collected and, after removal of soil particles, roots were agitated in 20 ml of SDW in a flask. Samples of 10-fold serial dilutions were plated onto TSA containing appropriate antibiotics, as above. CFUs were counted after 1–3 days.

### Assessing the Antifungal Capacity of Root Exudates

An *in vitro* assay was developed to test the antifungal capacity of root exudates (**Figure 2A**). Pepper seeds (cv. Bukwang) were prepared as described above. After 7 days of germination at 25°C, seeds were transferred to Petri dishes (diameter = 20 cm) and allowed to grow vertically. Plates were sealed with Saran wrap to retain moisture, and were half covered with aluminum foil (Daihan Eunpakgy Ind. Co., Ltd., Suwon, South Korea) to reduce exposure of roots to light from growth cabinets, which were set at 24 h light, 25°C (Vision Bio Tech., Seoul, South Korea). Four days after transplanting, 5 ml of 2,3-butanediol (1 mM or 1 μM) was drench-applied to the root system. Drenches with 1 mM benzothiadizole (BTH), which was commercialized SAR trigger by Syngenta as Actigard in the USA and BION in Europe and water were used as positive and negative controls, respectively. After cultivation for 2 days on potato dextrose broth agar (PDA, Becton, Dickinson and Company, Sparks, MD, USA) at 30°C, fungal spores were collected and their concentration was estimated using a hemocytometer. A sterilized cotton swab was used to inoculate the pepper root system with 10^5^ CFU/ml fungus, avoiding direct contact with the root surface. To determine any inhibitory effects of the root exudate on fungal growth, growth of fungal mycelium was imaged daily for a week after spore inoculation using a digital camera (Nikon Coolpix 4500, Japan). Fungus-free zones around the pepper roots were measured, and the mean was calculated (*n* = 20). The experiments were repeated three times.

### ITS-Based Fungus Identification

Fungus was isolated from pepper rhizosphere during assessing population density of *B. subtilis.* The fungus was cultured on the Poate Dextrose Broth agar (TSA, BactoTM, BD, Sparks, MD, USA) Total genomic DNA was extracted from the purified isolates using AccuPrep^®^ Genomic DNA Extraction Kit (Bioneer, Daejeon, South Korea). The nuclear ribosomal internal transcribed spacer (ITS) region of genomic DNA was amplified with ITS1 (5′-TCCGTAGGTGAACCTGCGG-3′) and ITS4 (5′-TCCTCCGCTTATTGATATGC-3′) primers using Quick PCR Premix containing Taq DNA polymerase, dNTPs, reaction buffer, and tracking dye (Genenmed, Daejeon, South Korea). PCR analyses were conducted in a PTC100 Thermal Cycler (MJ Research, Watertown, MA, USA) using an initial denaturation step of 95°C for 5 min; followed by 29 cycles of denaturation for 1 min at 94°C, primer annealing for 30 s at 52°C, and extension for 30 s at 72°C; with a final extension for 10 min at 72°C. Amplified PCR products were detected by electrophoresis on a 0.75% agarose gel, and purified with AccuPrep^®^ PCR Purification Kit (Bioneer, Daejeon, South Korea). The ITS region of the yeast isolates was sequenced using the same PCR primers and the ABI3700 automated DNA sequencer (Applied Biosystems, Foster City, CA, USA). The obtained sequence was submitted to NCBI^1^ for identification of the fungus.

### Collection of Root Exudates after Induction by 2,3-Butanediol Treatment

A new protocol was developed to collect root exudates from pepper seedlings. Seeds were germinated as described above, then positioned between two sterile filter papers (diameter = 120 mm) in a Petri dish (diameter = 150 mm and height = 20 mm). MS broth (10 ml) was applied to the Petri dish, which was then positioned vertically in an incubator at 25°C. After 4 days of incubation, excess MS broth was removed. A further 15 ml of MS broth containing 1 mM 2,3-butanediol, 1 μM 2,3-butanediol, or 1 mM BTH was drench-applied to filter papers. Treated Petri dishes were sealed with Saran wrap and partially covered with foil, as described above. Petri dishes were incubated at 25°C for a further 7 days before collection of root exudates. To collect root exudate in the hydroponic system, we modified our system previously described (**Figures 2D,F** inset; Song et al., 2015). Pepper seeds were surface-sterilized and germinated, as described above. Four-days-old seedlings were transferred to plates (60 mm × 15 mm, SPL) containing 26 ml of 0.5X MS liquid media. Plates were placed in the plastic container (phytohealth, 103 mm × 78.6 mm, SPL). 2 mM 2,3-butanediol, BTH and water control treatments were applied to plants as described above. The root exudates were collected at 7 and 14 days after treatments. For each replicate, containing 16 plants, 80 ml of root exudate was collected from plates. No media contamination was observed in the entire experiment.

### Assessment of Root Exudates on Bacterial Growth

A 96-well based assay was used to assess the effects of root exudates on bacterial growth. *B. subtilis* strains 168, 2,3-B(-), and 2,3-B(++) were cultured in TSB containing antibiotics as detailed above, then washed three times in 0.8% NaCl solution. Bacterial growth was monitored in 150 μl volumes containing TSB and root exudate (1:1 ratio). Optical density was determined every 15 min using a Bioscreen C system (Fluoroskan; Labsystems, Helsinki, Finland) at 30°C with continuous shaking.

### *Bacillus* Growth on the Different pH

The phenotype analysis for different pH was carried out by using a new tool, Phenotype MicroArrays (PMs). The 2,3-butanediol over-producer [2,3-B (++)] and non-producer [2,3-B (-)] were assayed on PM (Biolog) lane A1 to A12 of microplates PM10, testing different pH range from 3.5 to 10. PM technology uses the irreversible reduction of tetrazolium violet to formazan as a reporter of active metabolism. All procedures were performed as indicated by the manufacturer and previous study (Zhang and Biswas, 2009). Strains were grown at 30°C on BUG agar (Biolog), and then, each strain was picked with a sterile cotton swab from the agar surface and suspended in 15 ml of inoculation fluid (IF-0; Biolog) until a cell density of 85% transmittance was reached on a Biolog turbidimeter. In order to inoculate microplates PM10, 1% tetrazolium violet (vol/vol) was added to the suspension and the mixture was inoculated (100 μl per well). The photo was taken at 24 h after bacterial inoculation.

### Quantitative RT-PCR

Expression analysis of 2,3-butanediol-elicited defense genes was performed using quantitative real-time polymerase chain reaction (qRT-PCR), as described previously (Yang et al., 2011). Expression of *C. annum* basic β-1,3-glucanase (*CaPR 2*), *1*-aminocyclopropane-1-carboxylic acid (*CaACC*), *Systemic Acquired Resistance 8.2* (*CaSAR8.2*), *phenylalanine ammonia* (*CaPAL*), *lipid transfer protein* (*CaLTP 1*), *glutathione S-transferases* (*CaGST*), and *basic class II chitinase* (*CaChi2*) was reported previously during the defense response (Marrs, 1996; Garcia-Pineda and Lozoya-Gloria, 1999; Jung and Hwang, 2000; Lee et al., 2001; Hong and Hwang, 2002; Park et al., 2002; Mateos et al., 2009; Mazourek et al., 2009). The following primers were used: 5′-TAGTGAGACTAAGAAAGTTGGACG-3′ (*CaSAR8.2* Forward; GenBank accession no. AF327570.1), 5′-AAGAGTGCATGCAGTATCACAAAG-3′ (*CaSAR8.2* Reverse), 5′-ATTGGACGATGGAAGCCATCACCAG-3′ (*CaChi2* Forward; GenBank accession no. AF091235.1), 5′-ATATTCCGAATGTCTAAAGTGGTAC-3′ (*CaChi2* Reverse), 5′-TTTTAGCTATGCTGGTAATCCGCG-3′ (*CaPR2* Forward; GenBank accession no. AF227953.1), 5′-AAACCATGAGGACCAACAAAAGCG-3′ (*CaPR2* Reverse), 5′-CTCTAGGAAGGTGCTGTGGTGTC-3′ (*CaLTP1* Forward; GenBank accession no. AF118131.1), 5′-ACGGAAGGGCTGATTTCGGATG-3′ (*CaLTP1* Reverse), 5′-TCCACAAAGGGTCATGGTTT-3′ (*CaGST* Forward; Gen Bank accession no. HQ010689.1), 5′-GCCCTCTTCAATGACAGGAA-3′ (*CaGST* Reverse), 5′-ATTCGCGCTGCAACTAAGAT-3′ (*CaPAL* Forward; GenBank accession no. EU61657 5.1), 5′-CACCGTGTAAGGCCTTGTTT-3′ (*CaPAL* Reverse), 5′-AGTGGCCTTCAACTCCTCAA-3′ (*CaACC* Forward; Gen Bank accession no. AJ011109.1), and 5′-TTCCGTTTGTGATCACCTCA-3′ (*CaACC* Reverse). Relative mRNA levels were cali brated and normalized to the level of *CaActin* mRNA (Gen Bank accession no. AY572427). As a control, to ensure that equal amounts of RNA were used in each experiment, *CaActin* was analyzed using the primers 5′-CACTGAAGCACCCTTGAACCC-3′ and 5′-GAGACAACACCGCCTGAATAGC-3′. Candidate priming genes were amplified from 100 ng of cDNA by PCR using an annealing temperature of 55°C. A Chromo4 real-time PCR system (Bio-Rad Laboratories, Hercules, CA, USA) was used to carry out qRT-PCR. Reaction mixtures (20 μl) contained 10 μl of 2× Bril-liant SYBR Green QPCR master mix (Bio-Rad Laboratories, Hercules, CA, USA), cDNA, and 10 pmol of each primer. The thermocycle parameters were as follows: 10 min at 95°C, followed by 45 cycles of 30 s at 95°C, 60 s at 55°C, and 30 s at 72°C. Conditions were determined by comparing threshold values in a series of dilutions of the RT product, followed by a non-RT template control and a non-template control for each primer pair. Relative RNA levels were calibrated and normalized to the level of *CaACT1* mRNA (GenBank accession no. AY572427).

### Statistical Analysis

Data were subjected to ANOVA using JMP software version 4.0 (SAS Institute, Cary, NC, USA). Significance of biological or chemical treatment effects was determined by the magnitude of the *F*-value at *P* = 0.05. When a significant *F*-value was obtained for treatments, separation of means was accomplished using Fisher’s protected LSD at *P* = 0.05. Results of repeated trials of each experiment outlined above were similar, and one representative trial of each experiment is reported.

## Results

### Effect of 2,3-Butanediol Production on Rhizosphere Competence of *Bacillus subtilis*

**FIGURE 1:**
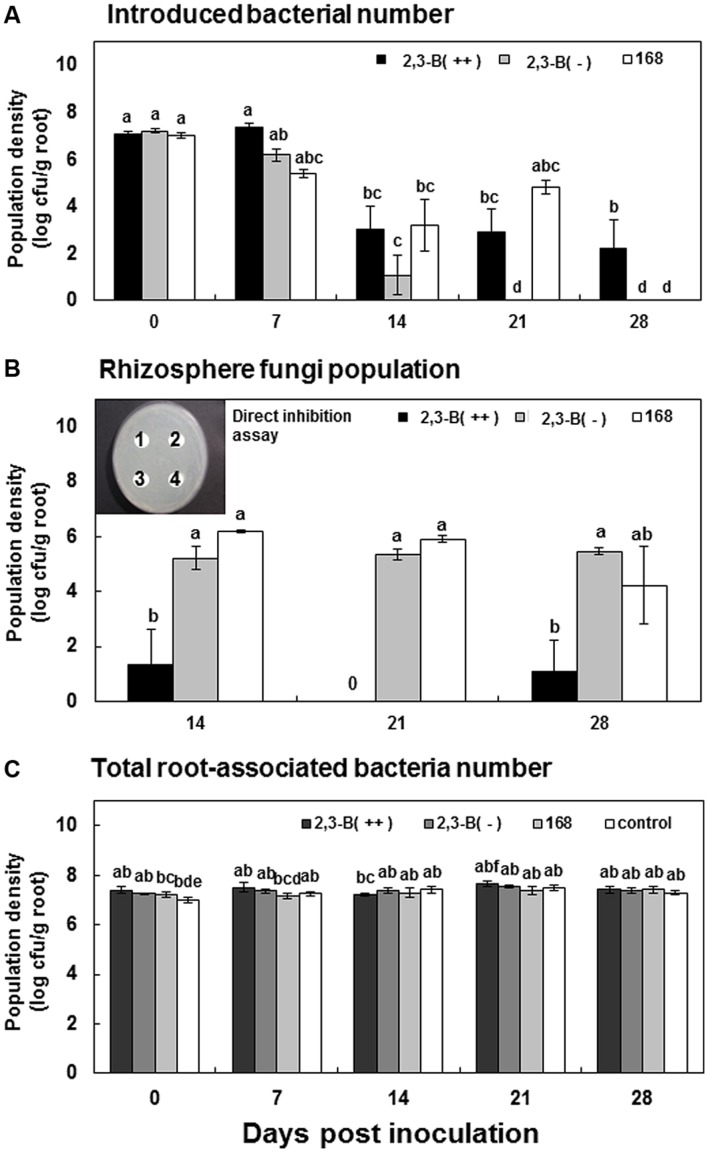
**The effect of 2,3-butanediol produced by *Bacillus subtilis* on bacterial rhizosphere competence.** Pepper roots were inoculated with 5 ml of bacterial suspension at 10^8^ cfu/ml (day 0). Pepper root samples were collected after removal of soil from roots. **(A)** Population densities of *B. subtilis* strains 168, 2,3-B(++), and 2,3-B(-) isolated from pepper roots 0, 7, 14, 21, and 28 days after inoculation. *B. subtilis* 168, the wild type; 2,3-B(++), 2,3-butanediol overexpression mutant derived from *B. subtilis* 168; 2,3-B(-), 2,3-butanediol null mutant derived from *B. subtilis* 168. **(B)** Fungal colonies on tryptic soy agar containing 100 μg/ml rifampicin recovered from pepper roots inoculated with strains 168, 2,3-B(++), and 2,3-B(-). **(C)** Direct inhibition test of 2,3-butanediol. 50 μl undiluted solution 1 (1), 10 fold diluted (2), and 100 fold diluted solution (3) 2,3-butanediol was spot-inoculated on the paper disks following spreading 10^5^ cfu/ml *Trichoderma* sp., spores. The 50 μl water treatment was used as control (4).

The role of bacterial volatile 2,3-butanediol *in situ* was examined in wild-type *B. subtilis* 168 and wild-type-derived null and overexpression strains. Wild-type *B. subtilis* 168 was previously shown to produce 2,3-butanediol (Ryu et al., 2004). A *pta-als* double mutant, 2,3-B(-), was unable to produce 2,3-butanediol, and the *pta* mutant 2,3-B(++) was an overproducer of 2,3-butanediol. Rifampicin resistance was generated in the three strains to allow selection from the pepper roots (data not shown). We hypothesized that the population densities of the three strains in the pepper rhizosphere at different time points (7 days intervals from inoculation) would differ. Total bacterial populations on pepper roots at inoculation were 10^7^–10^8^ cfu/g root and did not significantly differ between treatments (**Figure 1C**). Initial populations of each strain were ~10^7^ cfu/g root [7.0, 7.2, and 7.0 log cfu/g root for 2,3-B(++), 2,3-B(-), and 168, respectively]. After 7 days, populations were 7.3, 6.1, and 5.3 log cfu/g root for 2,3-B(++), 2,3-B(-), and 168, respectively. After 14 days, the population densities of 2,3-B(++) and 168 were 2.8- and 3-fold higher than that of 2,3-B(-), respectively (**Figure 1A**). Populations of strains 168 and 2,3-B(++) remained at pepper roots 21 days after inoculation, at 2.8 and 4.8 log cfu/g root, respectively (**Figure 1A**). Strain 2,3-B(-) was not found at pepper roots 21 days after inoculation. After 28 days, only strain 2,3-B(++) was present at pepper roots, at 10^2^ cfu/g root (**Figure 1A**). These results indicated that 2,3-butanediol facilitated maintenance of bacterial populations in the pepper rhizosphere. Unexpectedly, fungal colonies developed on the TSA plates used for isolation of *B. subtilis* from roots. The fungus was identified 98% as *Trichoderma* sp. from morphological characteristics and sequencing of the 18S ribosomal RNA ITS (data not shown). No fungal colonies were observed on isolation plates for 2,3-B(++) at 14, 21, and 28 days after inoculation (**Figure 1B**). Larger fungal populations were isolated from roots treated with strains 168 and 2,3-B(-) (**Figure 1B**). Fungal populations were 5.2, 5.3, and 5.4 log cfu/g root in the 2,3-B(-) treatment at days 14, 21, and 28, respectively (**Figure 1B**). For treatment with strain 168, fungal populations gradually decreased with time, at 6.1, 5.8, and 4.2 log cfu/g root at days 14, 21, and 28 respectively (**Figure 1B**). The number of *Trichoderma* sp. showed similar pattern when the repeated experiment was conducted. These results suggested that fungal growth could be directly inhibited by 2,3-butanediol. To test this, the fungus was challenged by pharmacological applications of 2,3-butanediol at different concentrations (10 μM–1 mM); however, no inhibition was observed, indicating that 2,3-butanediol did not directly affect fungal growth (**Figure 1B**). These results suggested that exposure of pepper roots to 2,3-butanediol might trigger the production of root exudates antagonistic to fungal growth.

### Indirect Effect of 2,3-Butanediol on Inhibition of Soil Fungus

**FIGURE 2:**
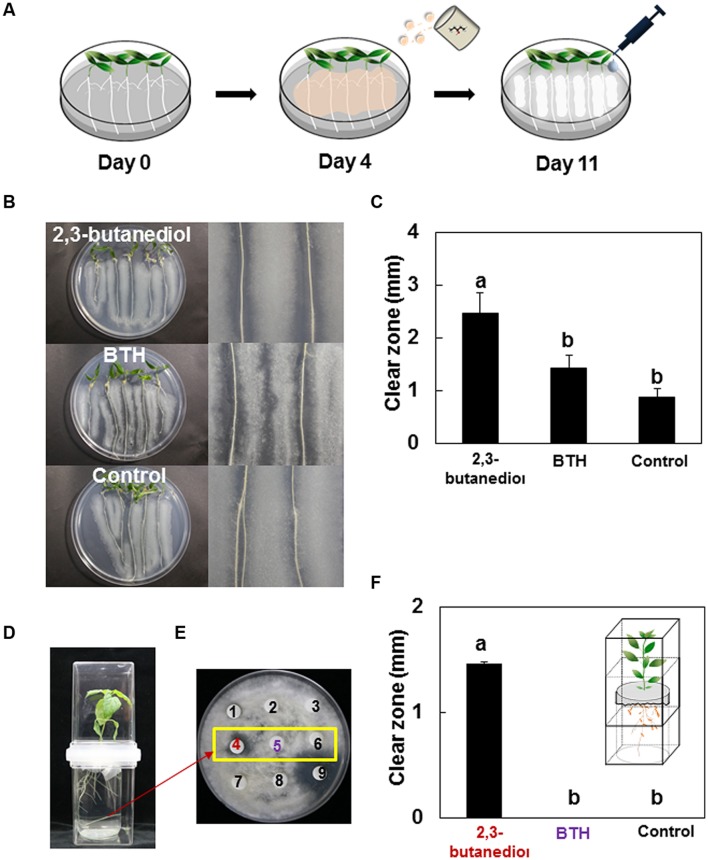
**Effect of 2,3-butanediol-elicited root exudates on fungal growth in the rhizosphere. (A)** Schematic of protocol for assessing fungal growth inhibition by root exudate elicited by 2,3-butanediol pre-treatment. Images in **(B)**, were taken 3 days after inoculation with *Trichoderma* sp. upon 1 mM 2,3-butanediol, 1 mM BTH, and water control. Left panels, whole pepper seedlings; right panels, magnified images of pepper roots. **(C)** Quantification of clear fungal inhibition zones surrounding roots. **(D)** Schematic of protocol for hydroponic system to collect root exudates by 2,3-butanediol pretreatment. **(E)** The fungal growth inhibition assay by root exudate. 1 = one time inoculation of root exudate treated with 2 mM 2.3-butanediol, 2 = one time inoculation of root exudate treated with 1 mM BTH, 3 = one time inoculation of root exudate treated with water, 4 = two time inoculation of root exudate treated with 2 mM 2.3-butanediol, 5 = two time inoculation of root exudate treated with 1 mM BTH, 6 = two time inoculation of root exudate treated with water, 7 = 2 mM 2.3-butanediol alone, 8 = 1 mM BTH alone, 9 = 50 μg/ml kanamycin as positive control **(F)**. Quantification of clear fungal inhibition zones by root exudates from root treated by 2,3-butanediol, BTH, and water treatments. Different letters above bars indicate significant differences between treatments as determined using LSD at *P* = 0.05.

To determine whether exudates of pepper roots treated with 2,3-butanediol contained antifungal agents, a novel protocol was devised in which seedlings were cultivated on Petri dishes, drenched with 2,3-butanediol, and inoculated with fungus (**Figure 2A**). Fungal growth was inhibited with all treatments (1 mM 2,3-butanediol, 1 mM BTH, and water; **Figures 2B–D**), with clear root inhibition zones of 2.45, 1.41, and 0.875 mm, respectively (**Figure 2E**). These results indicated that root exudates elicited by treatment with 2,3-butanediol and BTH inhibited the growth of soil fungus. We therefore wished to test whether root exudates elicited by 2,3-butanediol could also inhibit the growth of other microorganisms such as saprophytic and pathogenic soil bacteria. To obtain the clear evidence of antifungal capacity in the root exudate from pepper plant treated with 2,3-butanediol, the hydroponic system was set-up and successfully obtained enough root exudates. The three time inoculation of root exudate from 2,3-butanediol pre-treated root only showed clear zone (No. 4 in **Figures 2E,F**) while one time root or chemical alone treatments did not show any inhibitory effect (**Figures 2E,F**).

### Growth Kinetics of *B. subtilis* after Exposure to 2,3-Butanediol and Root Exudates

**FIGURE 3:**
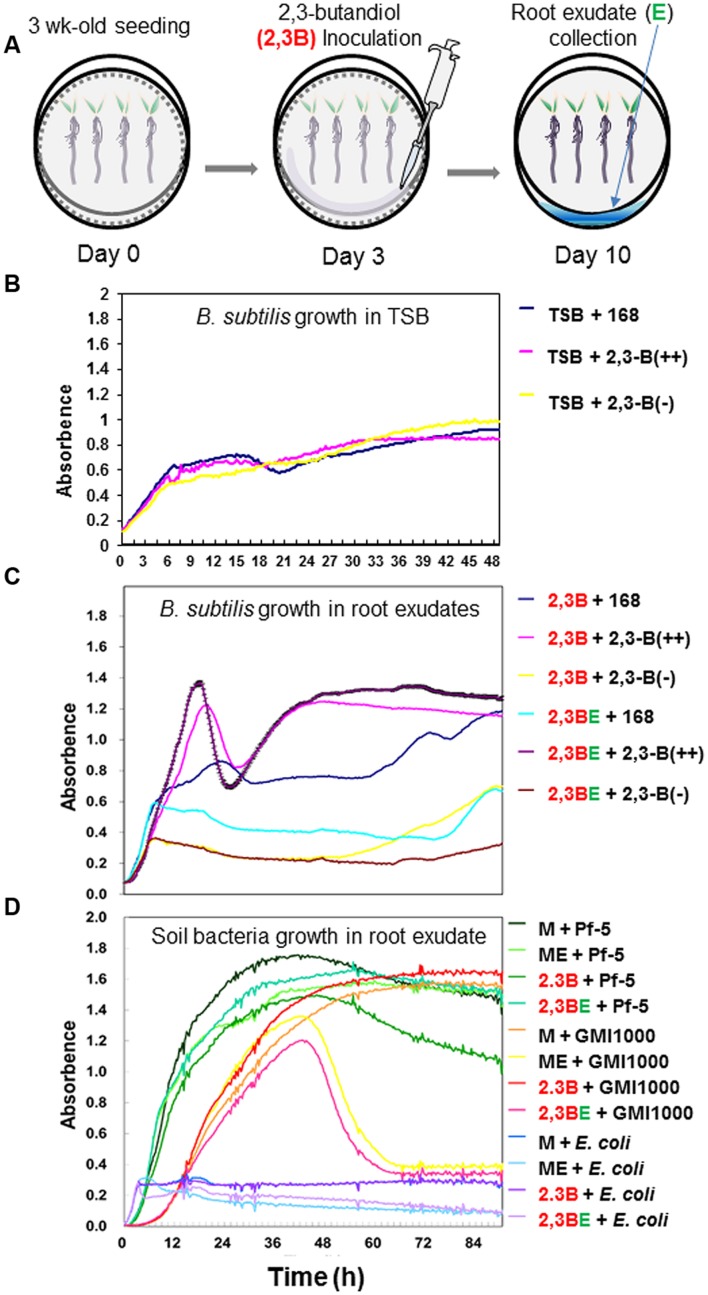
**Growth kinetics of *B. subtilis* wild-type and mutant strains after exposure to root exudates elicited by 2,3-butanediol.** Initial cell culture concentrations were OD_600_ = 0.02 and data are shown as log-normal plots. Tryptic soy broth supplemented with root exudate at a 1:1 ratio was applied to pepper roots. Aliquots (150 μl) from each culture were transferred to 100 wells of a Bioscreen plate. Plates were incubated in a Bioscreen C (Fluoroskan; Labsystems, Helsinki, Finland) with shaking at 30°C for ~4 days. The OD_600_ of each well was measured every 15 min. 2.3B = 2,3-butanediol; 23BE = root exudate collected from 2,3-butanediol-treated root system; 168 = *B. subtilis* 168; BSIP 1174 = *B. subtilis* BSIP 1174 (non-producer); BSIP 1171 = *B. subtilis* BSIP 1174 (overproducer); Pf-5 = *Pseudomonas protegens* Pf-5; GMI1000 = *Ralstonia solanacearum* GMI1000; M = MS broth; ME = MS broth amended with root exudate without treatment. **(A)** Schematic of protocol to extract root exudates after 2,3-butanediol application. **(B)** Growth kinetics of the three strains in control TSB medium. The figure indicate background expression of three strains 168, 2,3-B(++), and 2,3-B(-). **(C)** Growth of bacterial strains 168, 2,3-B(++), and 2,3-B(-) after treatment with 2,3-butanedol alone (referred to as 2,3B) or 2,3-butanediol-elicited root exudate (referred to as 2,3BE). **(D)** Growth kinetics of *P. protegens* Pf-5, *Ralstonia solanacearum* GMI1000, and *E. coli*. *P. protegens* Pf-5 is a non-pathogenic saprophyte that inhabits soil, water, and plant surface environments. Growth of *P. protegens* Pf-5 was not inhibited by 2,3-butanediol-elicited exudate. Growth of GMI1000, a soil-borne bacterial wilt pathogen, was inhibited by root exudates. *E. coli* was included as a bacterial control. Data shown are mean ± SEM of triplicate experiments.

To understand the role of root exudates (**Figure 3A**), and their effects on growth of *B. subtilis* strains 168, 2,3-B(++) (strain BSIP1171), and 2,3-B(-) (strain BSIP1174) and other soil-borne bacterial species, the growth kinetics of each treatment were assessed. *B. subtilis* strains 168, 2,3-B(++), and 2,3-B(-) had similar growth patterns on TSB medium, with maximum optical density of OD_600_ = 1 indicating that the mutation of the garget genes did not affect bacterial robustness under ideal growth condition (**Figure 3B**).

When exposed to root exudate from pepper root elicited by 2,3-butanediol treatment, growth of strains 168 (the wild type) and 2,3-B(++) (overexpression mutant) was less inhibited than that of 2,3-B(-) (null mutant; **Figure 3C**). After 2 days (48 h), growth of overexpresser strain 2,3-B(++) was higher when treated directly with 1 mM 2,3-butanediol (control) than when treated with exudates of 2,3-butanediol-treated pepper root (**Figure 3C**). Until log phase, the growth curve of strain 168 was similar between bacteria exposed to control and exudates (**Figure 3B**); nevertheless, after log phase, growth of control-treated 168 exceeded that of exudate-treated 168 (**Figure 3B**). Conversely, growth of exudate-treated 2,3-B(++) slightly exceeded that of control-treated 2,3-B(++) (**Figure 3C**). Furthermore, 2,3-B(-) was more sensitive to 2,3-butanediol itself while 2,3-B(++) was more resistant compared to wild type indicating that 2,3-butanediol non-producer can be less fitness than 2,3-butanediol producer (**Figure 3C**). The data suggested that 2,3-butanediol played an important role in protecting *B. subtilis* cells against harmful plant root exudates.

**FIGURE 4:**
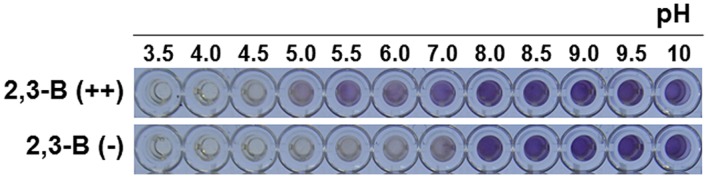
**Growth of *B. subtilis* 2,3-butanedol over-producer and non-producer under different pH condition.** The phenotype analysis for different pH was carried out by PM10 plate of Phenotype MicroArrays (PMs). The 2,3-butanediol over-producer [2,3-B (++)] and non-producer [2,3-B (-)] were assayed on PM (Biolog) lane A1 to A12 of microplates PM10, testing different pH range from 3.5 to 10. The photo was taken at 24 h after bacterial inoculation.

We next examined the effect of 2,3-butanediol on other soil bacteria, namely, the non-pathogenic biological control agent *Pseudomonas protegens* Pf-5, the bacterial wilt pathogen *R. solanacearum* GMI1000, and *E. coli*. Growth of the non-pathogenic saprophyte Pf-5 did not much affected by amendment of 2,3-butanediol-elicited root exudate (M + Pf-5, ME +Pf5, 2,3B +Pf5, and 2,3BE + Pf-5; **Figure 3D**). However, bacterial growth upon 2,3BE + Pf-5 gradually decreased after 60 h. In contrast growth of the pathogen GMI1000 was inhibited by exudates from pepper roots treated with 2,3-butanediol at 42 h after root exudate treatment (2,3B + GMI1000 vs. 2,3-BE + GMI1000). Although, the treatment ME (MS media solution plus root exudate without 2,3-butanediol treatment) also showed inhibitory effect on growth of strain GMI1000, the inhibition by root exudate collected from 2,3-butanediol treatment was greater (ME + GMI1000 vs. 2,3-BE + GMI1000). Growth of *E. coli* was totally inhibited by all treatment including control 2,3-butanediol treatments, TSB, MS, and root exudate mixtures (**Figure 3D**). These results indicate that 2,3-butanediol-elicited root exudate contains compounds that allow selective inhibition of bacterial growth depending on bacterial species. The bacterial growth upon different pH condition using by Phenotype Microarray system showed that the growth of 2,3-B(++) and 2,3-B(-) was indicated at pH 5 and pH 7 respectively (**Figure 4**). This results clearly showed that 2,3-butanediol production acts an important role on bacterial fitness under the acidic pH condition.

### Expression of Defense Genes in 2,3-Butanediol-Treated Pepper Roots

**FIGURE 5:**
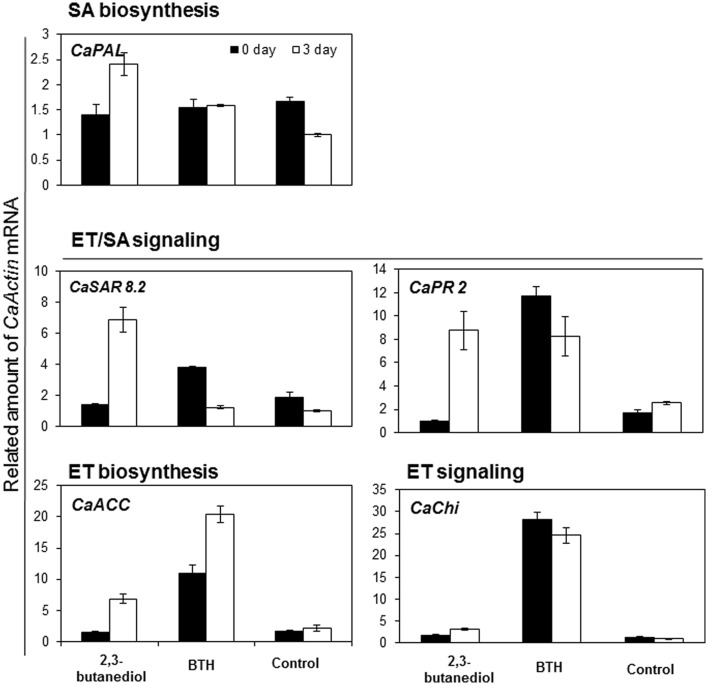
**Induction of defense genes in pepper roots after exposure to 2,3-butanediol.** Expression levels of nine defense genes were quantified by qRT-PCR. Expression of pepper defense-related genes, a salicylic acid (SA) biosynthesis gene *Capsicum annum phenylalanine ammonia* (*CaPAL*), two ethylene (ET) and SA signaling related genes *systemic acquired resistance gene 8.2* (*CaSAR8.2*) and *basic β-1,3-glucanase* (*CaPR 2*), an ET biosynthesis gene *1-aminocyclopropane-1-carboxylic acid* (*CaACC*), and an ET signaling-related *basic class II chitinase* (*CaChi 2*) were evaluated 0 and 3 days after *R. solanacearum* drench application at 7 days following. Bars indicate standard deviation (*n* = 4).

Induction of plant defense genes by 2,3-butanediol was assessed in pepper roots using qRT-PCR. At 3, 6, and 12 h after treatment, transcription of *basic β-1,3-glucanase* (*CaPR 2*) was higher in pepper roots treated with 1 mM 2,3-butanediol than in those treated with water (**Figure 5**). The effects of 2,3-butanediol on pathogen populations and gene expression in pathogen-challenged pepper roots were also assessed. Roots were exposed to the wilt pathogen *R. solanacearum* GMI1000 for 3 days, and bacterial populations were then determined. Fewer GMI1000 bacteria were recovered from roots treated with 1 mM and 10 μM 2,3-butanediol than from roots treated with water (**Table 1**). Root exudates of 2,3-butanediol-treated pepper were therefore able to inhibit growth of the bacterial pathogen GMI1000. Three days after pathogen challenge, expression levels of *Basic pathogenesis systemic acquired resistance gene 8.2* (*CaSAR8.2*) and *phenylalanine ammonia* (*CaPAL*) were higher in roots treated with 1 mM 2,3-butanediol than in positive control roots treated with 1 mM BTH (**Figure 5**). Conversely, expression levels of *1-aminocyclopropane-1-carboxylic acid* (*CaACC*), *lipid transfer protein* (*CaLTP 1*), and *basic class II chitinase* (*CaChi 2*) were lower in roots treated with 1 mM and 10 μM 2,3-butanediol than in positive control roots, but higher than in negative control roots treated with water (**Figure 5**). Transcription of *basic β-1,3-glucanase* (*CaPR 2*) was similar in roots treated with 1 mM and 10 μM 2,3-butanediol and roots treated with 1 mM BTH (**Figure 5**).

**Table 1:** Effect of 2,3-butanediol on elicitation of plant immunity against *Ralstonia solanacearum.*

Treatments	Pathogen population (x 10^7^ cfu/g root)	
	Day 0	Day 3
1 mM 2,3-butanediol	8.10^a^	8.35^a^
10 μM 2,3-butanediol	8.32^a^	8.26^a^
1 mM BTH	8.45^a^	7.92^a^
Control	7.96^a^	8.62^b^

*The bacterial cell count was measured at 14 days after pathogen inoculation. Different letters indicate significant differences between treatments (*P* = 0.05 according to least significant difference). The experiment was repeated three times with similar results (sample size, *n* = 5 replications per treatment).*

## Discussion

The first examinations of bacterial volatile-mediated plant growth and ISR, which used *B. subtilis* and *Arabidopsis thaliana* (Ryu et al., 2003, 2004), were followed by numerous studies examining the effects of bacterial volatiles on plants. Of the many volatile compounds identified from bacteria, 2,3-butanediol generated particular interest due to its broad spectrum effects on bacterial cells and induction of host responses (Ryu et al., 2004; Xiao and Xu, 2007; Rudrappa et al., 2010; Moons et al., 2011; Hahm et al., 2012). However, the impact of 2,3-butanediol on bacterial cells is yet to be elucidated. This prompted us to ask why bacteria, and soil bacteria in particular, might secrete 2,3-butanediol. Our results suggest that 2,3-butanediol promotes bacterial cell robustness against the effects of harmful compounds, such as root exudates (**Figures 1A** and **3C**). Both 2,3-butanediol and its precursor acetoin were shown to trigger ISR in plants (Han et al., 2006; Cortes-Barco et al., 2010a,b; Rudrappa et al., 2010; Hahm et al., 2012). Our study provides new information regarding the roles of 2,3-butanediol in root-associated bacteria *in situ*.

The volatile compound 2,3-butanediol is produced by many bacterial species as a result of a synthetic cascade, termed butanediol fermentation (Xiao and Xu, 2007). The exact role that butanediol fermentation plays in bacterial fitness is largely unknown. Classic literature suggested that 2,3-butanediol was formed to divert the cellular metabolism away from production of acidic compounds (Johansen et al., 1975). It was later discovered that 2,3-butanediol provided an alkaline environment during cell multiplication and protected bacterial cells against unfavorable acidic conditions, such as are found in eukaryotic hosts (Yoon and Mekalanos, 2006; Pradhan et al., 2010; Bari et al., 2011). Our results showed that a 2,3-butanediol null *B. subtilis* mutant was eliminated from the rhizosphere by 21 days after inoculation; however, corresponding wild-type and 2,3-butanediol overexpressing strains persisted for 21 and 28 days, respectively (**Figure 1A**). This can be hypothesized that 2,3-butanediol production by *B. subtilis* increased robustness of the acidic rhizosphere environment similar with rhizosphere microorganism (Huang and Chen, 2003; Hinsinger et al., 2003). Root exudates, which include acidic root secretion products, contribute to lowering the rhizosphere pH by releasing H^+^ or OH^-^ to compensate for unbalanced cation–anion uptake at the root surface (Hinsinger et al., 2003). In our system, the pH of root exudate was changed to 4.5 at 2 weeks after treatments compared to pH 5.8 at the beginning of experiment (data not shown). However, there are no difference upon pH between pretreatment of 2,3-butaendiol and water control. In addition to the proposed protective role of 2,3-butanediol in bacteria, 2,3-butanediol was recently shown to be critical for virulence of soft-rot plant pathogenic *Pectobacterium* spp. and *Dicheya* spp. Cell wall-degrading enzymes produced by the bacteria, such as protease, pectinase, and cellulose, require neutral pH for optimal function (Kwan et al., 2013). Bacterial robustness under acidic conditions and on normal artificial medium was compromised in a *Serratia plymuthica* budB mutant (Wevers et al., 2009).

In addition to the role of 2,3-butanediol as a bacterial protectant, 2,3-butanediol directly affects plant physiology and immunity (Han et al., 2006; Cortes-Barco et al., 2010a,b; Rudrappa et al., 2010; Hahm et al., 2012). In this study, the 2,3-butanediol null mutant could not stimulate plant defenses; however, wild-type *B. subtilis* successfully elicited a plant defense response against pathogens, indicating that bacterial 2,3-butanediol production played an important role in plant protection (Ryu et al., 2004; Rudrappa et al., 2010). No direct inhibition was observed when pathogenic bacteria and fungi were exposed to 2,3-butanediol, indicating that plant immunity rather than the direct effect of 2,3-butanediol provided inhibition (**Figure 1C**). Root application of 2,3-butanediol triggered root exudation and secretion of unknown compounds that differentially affected different species of bacteria in the rhizosphere. Exudate from roots treated with 2,3-butanediol suppressed growth of the soil-borne pathogen *R. solanacearum*, but enhanced growth of the saprophytic biocontrol bacterium *P. protegens* Pf-5 (**Figure 3D**). Our extra bioinformatics analyses revealed support the role of microbial production of 2,3-butanediol upon its robustness in rhizosphere: the *P. protegens* Pf-5 genome contains three major genes, acetolactate synthase (*alsS*), alpha-acetolactate decarboxylase (*alsD*), and 2,3-butanediol dehydrogenase (*bdhA*), needed for 2,3-butanediol production but *R. solanacearum* contains only the *alsS* gene with the result that less or no 2,3-butanediol can be produced (data not shown). Similarly, 2,3-butanediol null mutant of *B. subtilis* became more sensitive to pepper root exudates while the overexpressor of 2,3-butanediol was more resistance compared to wild type strain (**Figure 3C**). Moreover, 2,3-butanediol production help bacterial cells tolerate against acidic pH such as pH5 (**Figure 4**). Interestingly, the root exudate pH was stabilized as pH 4.5 in our hydroponic system indicating that *B. subtilis* may optimize the robustness using 2,3-butanediol production to acidification around root. Another possible explanation is that the two species may have different sensitivities to unknown compounds within the 2,3-butanediol-elicited root exudate. In our previous research, aboveground infestation of sucking insects like whitefly and aphids modulated the secretion of plant root exudates, leading to the recruitment of specific microbiota such as Gram-positive *Bacillus* spp. (Yang et al., 2011; Lee et al., 2012). Further research revealed that whitefly-infested tobacco plants secreted salicylic acid, which repressed *Agrobacterium tumefaciens* virulence genes and resulted in the suppression of crown gall formation (Song et al., 2015). We propose that plant defenses were induced by soil application of 2,3-butanediol, and that this induced the secretion of unknown compounds that targeted bacteria in a species-dependent manner. Detailed profiling of root exudates is required to characterize the compounds involved. The root exudate profiling was failed due to limitation to obtain large scale root exudates following 2,3-butanediol application.

Finally, one additional explanation is that different species may have different utilization capacities for root exudates. *P. protegens* Pf-5 is a saprophyte and encodes numerous enzymes that degrade organic materials, while *R. solanacearum* is a plant pathogen that primarily obtains nutrition from specific plant materials within xylem sap during the infection process (Salanoubat et al., 2002; Loper et al., 2007, 2012; Remenant et al., 2010).

The direct effect of 2,3-butanediol on pepper roots was demonstrated by changes in gene expression. Transcription of *CaPAL*, *CaSAR8.2*, *CaACC*, and *CaPR2* was affected when roots were drenched with 2,3-butanediol (**Figure 5**). This suggested that bacterial secretion of 2,3-butanediol activated plant defenses in the roots, mainly via salicylic acid and ethylene signaling pathways. This is supported by recent research in which direct soil application of 2,3-butanediol stimulated defense responses against foliar pathogenic anthracnose fungus and *Pseudomonas syringae* (Han et al., 2006; Cortes-Barco et al., 2010a,b). Previous studies showed 2,3-butanediol to be a signaling molecule involved in activation of immune responses in animal hosts. In mammals, 2,3-butanediol produced by pathogenic bacteria was closely associated with lung infections, including those caused by *Klebsiella pneumonia*, *Staphylococcus aureus*, and *Serratia marcescens.* Under these conditions, 2,3-butanediol produced an anti-inflammatory effect via inhibition of NF-κB signaling (Hsieh et al., 2007). Taken together, these data suggest that 2,3-butanediol is highly important for host colonization.

In summary, this study demonstrates that the bacterial volatile 2,3-butanediol has two key roles in the rhizosphere. First, 2,3-butanediol-elicited root exudates selectively affect different bacterial species, and, secondly, 2,3-butanediol protects bacterial cells against putative harmful plant root exudates and low pH. To our knowledge, this study is the first to demonstrate the significance of 2,3-butanediol on bacterial robustness *in planta*.

## Author Contributions

H-SY, Y-RA, S-YG, and C-MR conceived and designed research. H-SY, Y-RA, GCS, GL, and C-MR conducted all experiments. The manuscript was written by H-SY, Y-RA, and C-MR and approved by all other authors. SL conducted the pH sensitivity and provide **Figure 4**. The **Figure 4** was input during revising the manuscript.

## Conflict of Interest Statement

The authors declare that the research was conducted in the absence of any commercial or financial relationships that could be construed as a potential conflict of interest.

## Footnotes

1 http://blast.ncbi.nlm.nih.gov/Blast.cgi?PAGE_TYPE=BlastSearch&BLAST_SPEC=TargLociBlast
